# Supplementary material for: Pilomatricoma in Syndromic Contexts: A Literature Review and a Report of a Case in Apert Syndrome
Source: Dermatopathology (Basel). 2025 Aug 1;12(3):24. doi: 10.3390/dermatopathology12030024 (PMC12371956; doi:10.3390/dermatopathology12030024)
Supplement: Supplementary file 1 [file dermatopathology-12-00024-s001.zip › dermatopathology-3616399-supplementary.pdf]

Check-list PRISMA

TITLE

Item	Description	Detail
1	The title clearly identifies the work as a systematic review.	*Pilomatricoma in syndromic contexts: A Literature Review and a report of a case in Apert Syndrome*

ABSTRACT

Item	Description	Detail
2	The abstract follows a structure compliant with PRISMA for Abstracts.	Narrative

INTRODUCTION

Item	Description	Detail
3	Rationale.	The introduction clearly highlights the need to fill a gap in knowledge regarding the association between pilomatricoma and genetic syndromes.
4	Objectives.	The aim of this review is to summarize the literature on pilomatricomas in genetic syndromes and to present the first reported case in a patient with Apert syndrome.

METHODS

Item	Description	Detail
5	Eligibility criteria.	Clearly defined: exclusion of sporadic cases, duplicates, and cases without patient data.
6	Information sources.	Stated: PubMed and Cochrane; last accessed in February 2024.
7	Search strategy.	Search terms listed in the “Methods” section.
8	Study selection process.	Detailed process with PRISMA flow diagram included.
9	Data collection process.	Study selection and data extraction were performed independently by two reviewers, with discussion in case of disagreement.
10a	Data items – outcomes.	Collected variables: age, sex, syndrome, genetic mutations, number and location of pilomatricomas.
10b	Data items – other variables.	Secondary data described (genetic mutations, clinical features).

Item	Description	Detail
11	Study risk of bias assessment.	No formal risk of bias assessment was performed, as all included studies were case reports or case series for which no validated tools exist.
12	Effect measures.	As the data consist of descriptive case reports without comparable quantitative outcomes, effect measures are not applicable.
13	Synthesis methods.	13a: Structured narrative synthesis with tabular summary. 13b: No meta-analysis. 13c: Methods for heterogeneity, sensitivity, and meta-regression not applicable.
14	Reporting bias assessment.	No assessment of publication bias was performed, since the review only included published case reports, whose selection is influenced by rarity rather than quantitative data.
15	Certainty assessment.	No formal certainty assessment (e.g., GRADE) was applied due to the anecdotal nature of the data and the observational design of included studies.

RESULTS

Item	Description	Detail
16	Study selection (PRISMA flowchart).	Complete description and PRISMA flow diagram included.
17	Characteristics of included studies.	All studies cited and tabulated with key characteristics (Tables 1 and 2).
18	Risk of bias in included studies.	No formal risk of bias assessment was conducted, since the included studies were exclusively case reports and case series, for which validated tools do not exist.
19	Results of individual studies.	Described in the results section and summarized in Table 2.
20	Results of syntheses.	20a: Narrative description based on syndrome, mutation, and lesion number.
21	Reporting biases.	No assessment of publication bias was performed, as the review consists exclusively of narrative case reports and series.
22	Certainty of evidence.	The certainty of evidence was not assessed, as the data derive exclusively from anecdotal observational reports.

DISCUSSION

Item	Description	Detail
23a	Interpretation.	The discussion is contextualized with the existing literature.
23b	Limitations of evidence.	Small sample size, observational nature, potential selection bias.
23c	Limitations of review process.	No risk of bias or certainty assessments were conducted. The review was not registered and no protocol was developed.
23d	Implications.	Pilomatricoma may serve as an early clinical indicator of an underlying genetic syndrome.

OTHER INFORMATION

Item	Description	Detail
24	Registration and protocol.	This review was not registered in a public database (e.g., PROSPERO), and no protocol was developed.
25	Support.	No funding received.
26	Competing interests.	No conflicts of interest declared.
27	Availability of data, code and other materials.	Data analyzed are available upon request from the corresponding author.
